# Carbon Monoxide Poisoning: The Great Imitator

**DOI:** 10.51894/001c.6343

**Published:** 2017-08-24

**Authors:** Christopher Velasquez, Tye Patchana, Brian McParland, Jonathan Lovy, Ahmad Maarouf, Christopher Whitty

**Affiliations:** 1 Michigan State University College of Osteopathic Medicine, East Lansing, MI. Beaumont Hospital Southshore Campus, Trenton, MI.; 2 Lake Erie College of Osteopathic Medicine, Erie, PA. Beaumont Hospital Southshore Campus, Trenton, MI.; 3 Beaumont Hospital Southshore Campus, Trenton, MI. Beaumont Hospital Internal Medicine Residency Program Director Southshore Campus, Trenton, MI.; 4 Beaumont Hospital Southshore Campus, Trenton, MI. Beaumont Hospital Department of Critical Care Medicine Southshore Campus, Trenton, MI.; 5 Beaumont Hospital Southshore Campus, Trenton, MI. Michigan State University Department of Neurology & Ophthalmology, East Lansing MI.

**Keywords:** carboxyhemoglobin, basal ganglia, globus pallidus, carbon monoxide

## Abstract

Carbon Monoxide (CO) is one of the leading causes of poison deaths in the United States. Signs and symptoms are clinically variable secondary to inconsistent targeting of highly metabolic tissues by the gas. We report a case of a man in his early to mid-30’s presenting to the emergency department with mental status changes, fatigue, headache, and flu-like symptoms for three days. The patient had been working on his motor vehicles in the garage during this time, using a portable diesel powered space heater to keep warm. Subsequent neurology and cardiology workup demonstrated bilateral globus pallidus (GP) lesions on brain imaging, increased non-myocardial infarction troponin levels, carboxyhemoglobin (COHb) level of 3.8%, elevated liver enzymes, and acute kidney failure. In this setting of his delayed presentation as a smoker with carbon monoxide poisoning, carboxyhemoglobin levels alone become less reliable. This report investigates the use of bilateral GP lesions, the most frequently affected structure, as well as damage preference to highly metabolic tissues to assist in diagnosis and prognosis for CO poisoning. Our observations can be used for further study of the relationship between bilateral GP necrosis and initial presentation and outcome of patients experiencing CO poisoning leading to earlier recognition, treatment, and decreased morbidity/mortality.

## INTRODUCTION

Carbon monoxide (CO) is the most common cause of death from accidental poisoning in the world.^1^ Although the United States Center for Disease Control and Prevention (CDC) states that there are approximately 500 accidental non-fire related CO poisoning deaths every year, other estimates put this number as high as 1,000-2,000 annual cases.^2^ In addition, CO poisoning in responsible for approximately 50,000 annual emergency room visits.^3^

However, the estimated reporting of CO-related morbidity and deaths is likely underreported since the United States does not have a comprehensive national system of CO surveillance.^4^ In addition, misdiagnosis can occur due to variable clinical presentations and nonspecific signs and symptoms.^4^ The variability and nonspecific symptomatology is largely due to the colorless, odorless features of CO gas combined with the nature, severity, and duration of exposure.^5^

Exposures most frequently occur in winter months due to smoke inhalation from fires, motor vehicle exhaust, or the burning of fuel (e.g., oil, wood, coal, gasoline, natural gas) in poorly functioning or improperly ventilated devices (e.g., heating systems, stoves, charcoal grills, portable generators, electrical heaters, etc.). In this report, we present a severe case of CO poisoning that demonstrates how brain imaging may provide an essential clue during provider diagnosis and prognosis of exposures.

### Case Description

A Caucasian male in their early to mid 30’s presented to an emergency department (ED) by ambulance with mental status changes. The individual was a smoker without history of medical problems. However, providers suspected drug overdose due to his history of suboxone and hydrocodone use in the past and he received 8 mg. of Narcan prior to arrival without improvement. Initial pulse oximetry was especially low in the 50% range and he had been treated by paramedics with high-flow oxygen via nonrebreather mask.

His wife reported that the patient had experienced increased drowsiness and fatigue during the past three days, spending most of his time lying on the couch with associated flu-like symptoms and headache. The patient's wife was unable to wake the patient from sleep on the day of his presentation, even after splashing a bucket of cold water onto his face, prompting her to call 911. He had also had one episode of vomiting enroute to the ED.

Upon his arrival, the patient had a low level of consciousness (Glasgow Coma Scale of 3) and his initial vital signs included a temperature of 97.4^o^ F, blood pressure of 96/49, heart rate of 110, respiratory rate of 25 breaths/min and an improved pulse oximetry reading of 96%. He was immediately intubated to maintain his airway. Physical examination was otherwise unremarkable. Hypoglycemia was absent, and his ECG was normal. An initial CT scan of the head was read as negative for acute intracranial pathology. Urine toxicology was also negative.

Due to the patient’s obesity, lumbar puncture was particularly difficult and multiple attempts in the ED were unsuccessful. Interventional radiology was consulted to perform lumbar puncture with cerebral spinal fluid (CSF) results pending. He was admitted to the Intensive Care Unit with respiratory isolation and his working differential diagnosis included meningitis, for which he was started on empiric antibiotics. Upon neurological consult and evaluation, additional history revealed that the patient had been working on several motor vehicles in the garage during the past week and using a portable diesel space heater. Upon reviewing laboratory studies, the patient’s combined acute liver failure (i.e., AST of 1144, ALT of 1692) and kidney failure (Creatinine of 2.05) indicated global hypoxia.

**Figure 1:**
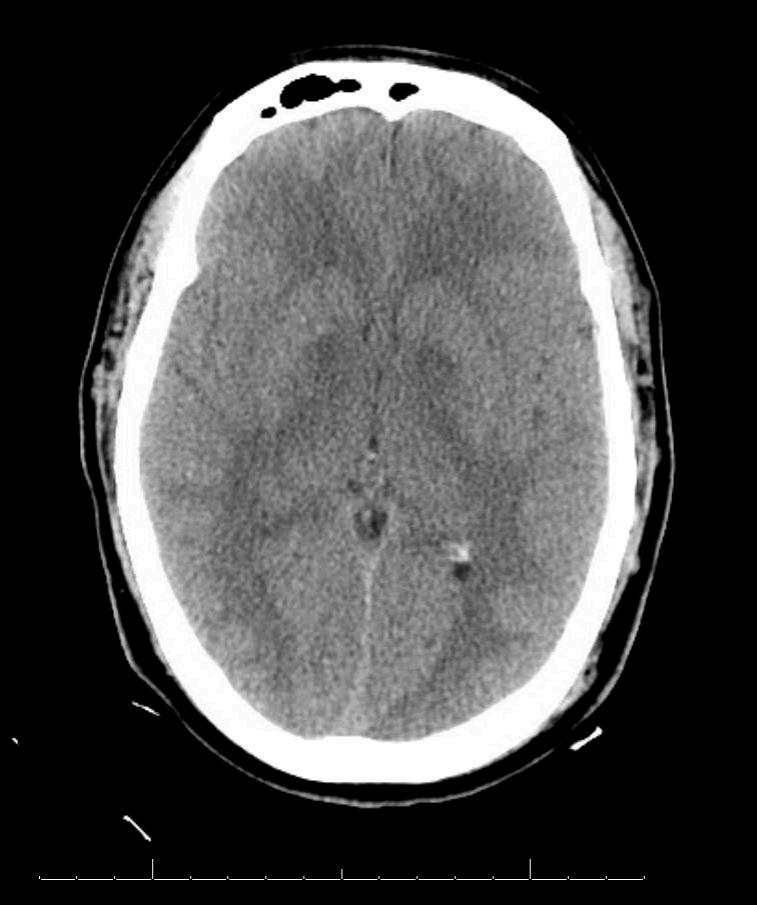
CT Head Without Contrast Demonstrating Symmetric Hypodensity of Bilateral Globus Pallidus (GP)

**Figure 2:**
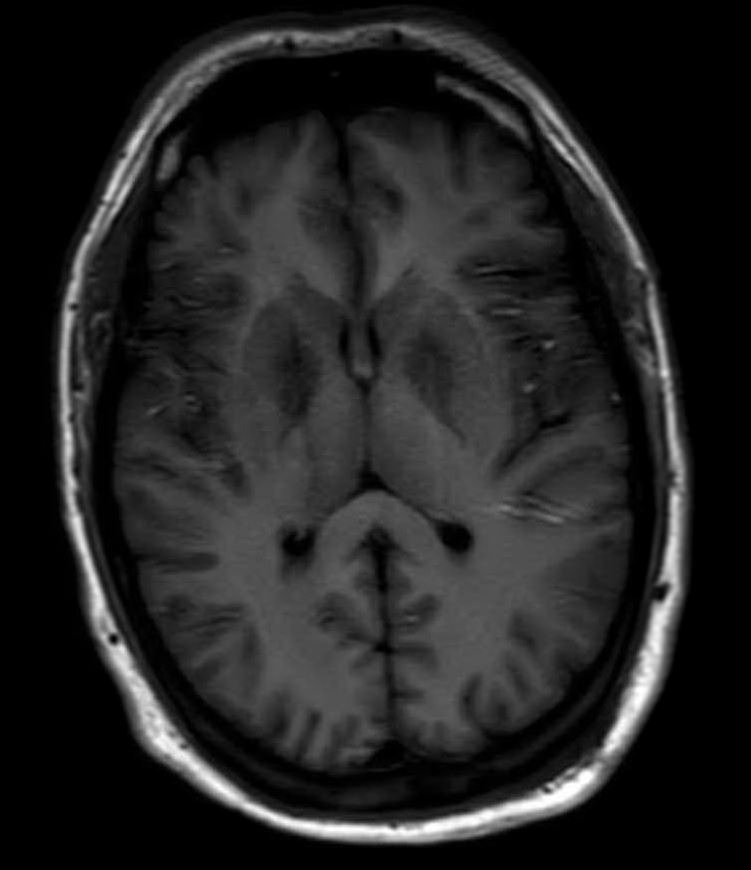
T1 FLAIR MRI Brain Showing Hypointense GP Relative to Surrounding Brain Parenchyma

**Figure 3:**
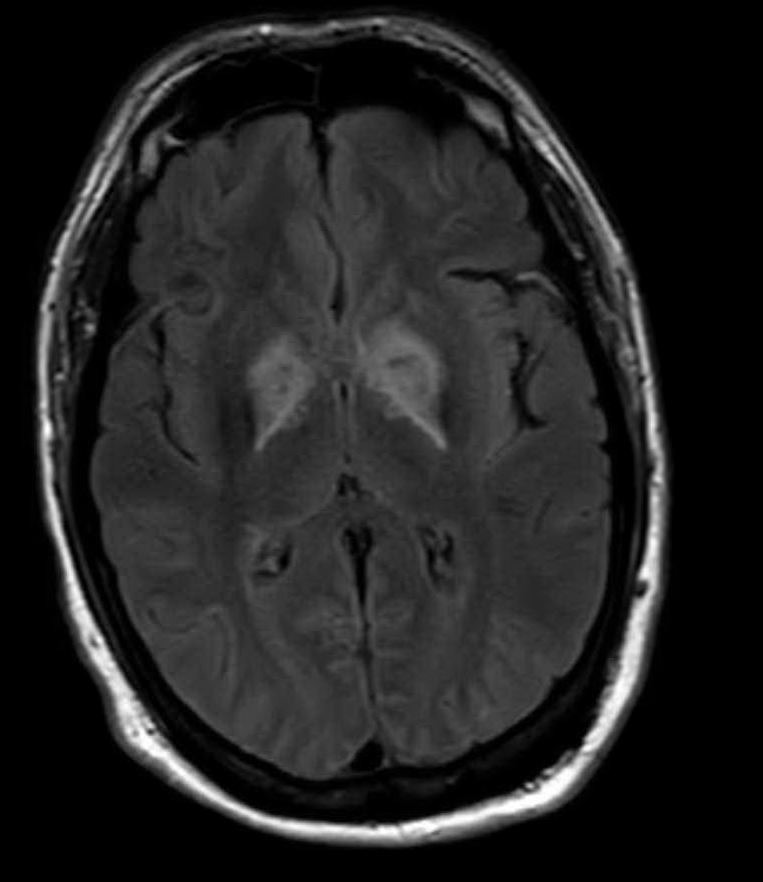
AXIAL T2 FLAIR MRI Brain Showing Hyperintense GP Relative to Surrounding Brain Parenchyma

Further reevaluation of the patient’s initial head CT noted bilateral hypodensities in the globi pallidi (Figure 1). An MRI was ordered, revealing restricted diffusion on diffusion weighted imaging with symmetric bilateral involvement of the globi pallidi (GP). Hypointensity to surrounding brain parenchyma was noted on T1 Axial MRI, with corresponding hyperintensity on T2 Axial MRI. MRI films are included in this report (Figures 2 and 3). An arterial blood sample revealed a carboxyhemoglobin (COHb) level of 3.8% (above the absolute upper normal limit of 3% for smokers). Per his wife, the patient had smoked one to two packs of cigarettes daily for the past 18 years.

The lumbar puncture performed by interventional radiology revealed no active evidence of meningitis. Blood, sputum, CSF, and urine cultures remained negative throughout hospitalization. On hospital Day 4, the patient was extubated but remained obtunded.

On Day 5 of his hospital stay, the patient became alert and oriented and a more complete review of systems was able to be obtained. The patient complained of fevers, chills, cough, and global myalgia. He was restless and could not find a comfortable position at night. His respiratory rate had continued to be increased (29/min). He remained on Zosyn for aspiration pneumonia coverage and his liver enzymes began to normalize (i.e., AST 65, ALT 749). An electroencephalogram (EEG) that had been ordered by the Neurology service showed slowed waveforms with no seizure activity.

Cardiology consult following a non-MI troponin increase (0.19) agreed with the assessment of CO poisoning and advocated for continued supportive care. The patient was moved on Day 5 from the intensive care unit to the general medical floor. He was discharged home two days later.

## DISCUSSION

CO is an odorless, tasteless, and invisible gas produced by incomplete combustion of carbon containing compounds. Exposures are most frequently seen during the winter months in cold climates due to accidental smoke inhalation from fires or use of gasoline powered portable generators, space heaters, or camp stoves in poorly ventilated areas. Providers should also consider the possibility of an intentional CO exposure during a suicide attempt. Exposure in the summer months most commonly occurs from boats (i.e., primarily from engine or generator exhaust). Exposures are also more common after natural disasters such as hurricanes or floods due to the use of portable fuel and electricity for heating, cooking, and cleanup. For example, there were 51 cases of CO poisoning reported after Hurricaine Katrina in 2005 by hyperbaric oxygen facilities in Alabama, Louisiana, and Mississippi.^6^

Since CO is inhaled, it diffuses across the alveolar membrane and into the pulmonary capillaries to form carboxyhemoglobin (COHb) inside red blood cells. Since the most common cellular target of CO is heme, it tends to bind to heme-containing proteins including hemoglobin, cytochrome C, cytochrome P450s, and myoglobin.^7,8^ Oxygen delivery to tissues is reduced and the tissues at greatest risk are those with high metabolic demands which may result in global hypoxia as exemplified in our patient.

This gas also disrupts ATP production by binding Cytochrome C Oxidase in cardiac myocyte mitochondria as reflected in this man’s non-MI related troponin increase. Interestingly, several studies report that CO-poisoned patients presenting with acute cardiac injury have significantly higher long-term mortality and neurologic sequelae.^9,10^ For this reason, it is important for providers to obtain an ECG and cardiac biomarkers to identify myocardial injury in the workup of severe CO poisoning.

It is also significant to note that there is no clear relationship between CO-oximetry measured COHb levels with symptoms.^11^ The first and overall most common symptom is headache, as the brain is most sensitive to CO poisoning. Beyond classically known signs and symptoms (i.e., headache, fatigue, flu-like/viral illness, nausea, vomiting, dizziness, shortness of breath, chest pain, and pink or cherry-red skin), CO poisoning has been associated with increased long term morbidity & mortality.^12^ Specifically, patients with CO poisoning have been shown to have three-fold increases in mortality compared with matched, unexposed individuals at a median post-exposure follow-up period of 7.6 years.^10^

In the appropriate clinical context, a serum COHb level over 3% for nonsmokers and over 15% in smokers per arterial or venous blood confirms the diagnosis of CO poisoning. However, levels may be low if the patient has already received supplemental oxygen or if delay occurs between exposure and testing. Pulse oximetry is also inaccurate because of the similar absorption characteristics of oxyhemoglobin and carboxyhemoglobin. Combined with nonspecific sign and symptom presentation, it can be very difficult to make a firm diagnosis of CO poisoning, resulting in increased morbidity and mortality and underreporting of the deadly disease.

When attempting an earlier recognition of CO poisoning, one option may be to explore the subtle and often overlooked neuroimaging findings that continue to be helpful in narrowing down this difficult diagnoses, particularly in cases of encephalopathy where patient history is not attainable. Providers need to conside that other specific toxins (e.g., methanol, lead, etc.) can selectively affect distinct areas of the brain.^13–15^

The GP, a deep subcortical structure at the inferior base of the brain, is the most frequently affected structure in CO poisoning and usually damaged immediately, symmetrically, and bilaterally.^16^ The leading theory for why CO specifically targets the GP is that it contains the highest iron content in the brain.^17^ The ischemic CO induced lesions in the bilateral GP of the basal ganglia can be seen as symmetric hypodensity on CT, hyperintensity on T2, hypointensity on T1, and restricted diffusion on diffusion weighted imaging MRI. The patient described in this case report demonstrated these classic findings on imaging that ultimately confirmed the diagnosis (Figures 1, 2, and 3).

The cornerstone treatment of CO poisoning is administration of high flow oxygen via non rebreather mask regardless of the pulse oximetry readings. Hyperbaric oxygen therapy has continued to be a controversial intervention and only one high quality randomized trial has indicated its use resulting in improved neurological outcomes at 12 months.^18^ Additionally, authors of a recent retrospective review from Taiwan evaluating 25,757 patients with CO poisoning demonstrated a survival advantage in patients treated with hyperbaric oxygen.^19^ However, there is currently no absolute indication and standardization for hyperbaric oxygen therapy and further randomized controlled trials are needed. As of January 2017, the American College of Emergency Physicians has only assigned a level B recommendation for the use of hyperbaric oxygen therapy.^20^

CO exposure may be prevented by advocating CDC recommendations to install a battery-operated or battery back-up CO detector in patients' homes, basements and garages, checking/replacing the battery when changing clock settings each spring and fall. Since CO is slightly lighter than air, carbon monoxide detectors have historically been placed on the ceilings or high up on a wall. However, CO can mix and diffuse in its environment and detectors that are placed lower on the wall are generally adequate.^12^

## CONCLUSIONS

Carbon monoxide poisoning is a complex clinical condition due to its variable clinical presentation and nonspecific symptoms. It is therefore crucial to obtain a detailed history and keep this diagnosis high on the differential in any patient with headache and/or mental status changes. Although always difficult in an unstable patient presenting to the ED, a detailed focused history will remain the foundation in practicing medicine.

Especially during the winter months, primary care providers should ask specific questions to obtain historical information concerning space heater or generator use, work in enclosed spaces such as a garage, prior suicide attempts, or multiple family members presenting at once with similar complaints. As one of the leading causes of poison deaths in the United States, CO poisoning requires that providers in all healthcare settings recognize its prevalence, presentation, and treatment. Unfortunately, carbon monoxide poisoning classically presents with nonspecific symptoms and current diagnostic modalities including carboxyhemoglobin levels are often unreliable as indicated in this case report.

Perhaps this diagnosis may be more consistently revealed when a suspicious history is combined with findings of symmetric bilateral lesions of the GP on brain imaging. Future research is required to focus on the diagnostic and prognostic usefulness of neuroimaging which may ultimately identify novel interventions for CO poisoning victims.

### Conflict of Interest

The authors declare no conflict of interest.
